# Association between red blood cell distribution width and left ventricular hypertrophy in pediatric essential hypertension

**DOI:** 10.3389/fped.2023.1088535

**Published:** 2023-02-02

**Authors:** Xiaodong Sun, Yang Liu, Yanyan Liu, Hui Wang, Bo Liu, Lin Shi

**Affiliations:** ^1^ Capital Institute of Pediatrics, Beijing, China; ^2^ Department of Cardiology, Children's Hospital, Capital Institute of Pediatrics, Beijing, China

**Keywords:** essential hypertension, pediatrics, red blood cell distribution width, left ventricular hypertrophy, target organ damage

## Abstract

**Aim:**

Left ventricular hypertrophy (LVH) is one of the most common types of target organ damage in hypertension. The red blood cell distribution width (RDW) is closely related to many cardiovascular diseases, including hypertension. The aim of this study was to analyze the relationship between the RDW level and LVH in pediatric essential hypertension.

**Materials and methods:**

A total of 429 untreated children and adolescents with essential hypertension were recruited and divided into an LVH group (*n* = 114) and non-LVH group (*n* = 315) according to left ventricular mass index (LVMI) and relative wall thickness (RWT) by color Doppler ultrasound. Spearman correlation analysis was used to determine the relationship between RDW and LVMI, RWT. The effect of RDW on LVH was determined using a multivariate logistic regression analysis. To assess the predictive value of RDW on LVH, the receiver operating characteristic (ROC) curve was used.

**Results:**

The level of RDW in children with hypertension in the LVH group was significantly higher than that in the non-LVH group (13.0 [12.0, 13.0] vs. 12.4 [12.0, 13.0] %, *P* = 0.001). The incidence of low and high quantiles of LVH was 21.0% and 32.0%, respectively. Spearman correlation analysis showed that RDW was positively correlated with C-reactive protein (CRP), LVMI, RWT, and red blood cell (RBC) count (*P* all < 0.05), and negatively correlated with hemoglobin (HGB) level, mean corpuscular volume (MCV), mean corpuscular hemoglobin (MCH), and mean corpuscular hemoglobin concentration (MCHC) (*P* all < 0.05). After adjusting for various confounding factors, a multivariate logistic regression model revealed that RDW was an independent risk factor for LVH (OR = 1.946, 95% CI: 1.324–2.861, *P* = 0.001). The area under the ROC curve of RDW predicting centripetal hypertrophy was 0.700 (95% CI: 0.541–0.859, *P* < 0.05) in pediatric essential hypertension.

**Conclusions:**

Increased RDW levels are an independent risk factor for LVH in pediatric essential hypertension, and RDW may be a predictor of LVH in untreated pediatric essential hypertension.

## Introduction

In China, the prevalence of essential hypertension among children and adolescents aged 7 to 17 increased from 8.5% in 1991 to 19.2% in 2015, with an annual increase in the prevalence (1). Hypertension in childhood is closely related to subclinical target organ damage, the most common of which is left ventricular hypertrophy (LVH) (2, 3). Recent studies have shown that hypertensive children and adolescents with LVH may have an increased cardiovascular risk in adulthood (4, 5). As a result, identifying specific risk factors for LVH in pediatric essential hypertensive patients is critical for preventing long-term adverse cardiovascular events.

The red blood cell distribution width (RDW) is an index of the size heterogeneity of circulating red blood cells (RBCs) and is used to differentiate anemia. Recent studies have found that the RDW is closely related to morbidity and mortality of many cardiovascular diseases, including acute myocardial infarction, acute heart failure, atrial fibrillation, coronary artery disease, hypertrophic cardiomyopathy (6–10). Inflammatory responses and oxidative stress have been proposed as the mechanisms underlying the association of RDW with cardiovascular diseases morbidity and mortality (11). RDW research in the field of hypertension has attracted increasing scholars’ attention. Studies have shown that high RDW is independently associated with an increased risk of hypertension (12, 13). Moreover, RDW levels are higher in patients with prehypertension and hypertension than in individuals with normal blood pressure (14). The mechanisms explaining the relationship between RDW and hypertension include renin-angiotensin-aldosterone system (RAAS) activation and inflammatory responses. The RAAS system plays an important role in neurohumoral regulation through angiotensin II, which is recognized as one of the causes of hypertension (15). An increase in angiotensin II can directly promote EPO secretion and increase the production of immature RBCs, resulting in an increase in RDW (16, 17). Increased inflammatory cytokine levels alter iron metabolism and bone marrow function *in vivo*, as well as increase erythrocyte size inequality and RDW levels (8). Higher RDW levels impair blood flow and induce hypoxia, promoting endothelial dysfunction and raising blood pressure (18, 19). Simultaneously, angiotensin II and proinflammatory cytokines can cause cardiomyocyte hypertrophy, regulate left ventricular remodeling, and play a role in the formation and progression of LVH (20, 21). Some studies have discovered a link between RDW and LVH in hypertensive adults (22–24). However, no reports have been published on the relationship between RDW and LVH in pediatric essential hypertension. Therefore, the aim of this study was to evaluate the relationship between RDW levels and LVH in pediatric essential hypertension.

## Materials and methods

### Study population

This case-control study was retrospective and conducted at a single center, which was conducted at the Department of Cardiology, Children's Hospital Capital Institute of Pediatrics (Tertiary a-level hospital, Beijing, China). From January 2019 to June 2022, untreated children who were first diagnosed with hypertension were consecutively enrolled according to the following inclusion criteria: (A) age: 6–17 years old; (B) diagnosis and staging of hypertension were based on “2018 Chinese Guidelines for Prevention and Treatment of Hypertension” (25); (C) demographic data (sex, age), past medical history (any acute infection, blood transfusion, trauma, or surgery within one month before admission; whether there are hematological diseases, rheumatic autoimmune diseases, malignant tumors, serious cardiovascular diseases have not been cured), and family history (family history of hypertension) were collected, physical examination (height, weight), laboratory examination (including complete blood count and biochemical indicators), 24 h ambulatory blood pressure monitoring (ABPM) (24 h systolic blood pressure [24 h SBP], 24 h diastolic blood pressure [24 h DBP]), and echocardiography (left ventricular internal dimension [LVIDd], interventricular septal thickness [IVST], left ventricular posterior wall thickness [LVPWT], left ventricular ejection fraction [LVEF], fractional shortening [FS]) were completed during hospitalization, the above data were available and complete in the case system. The exclusion criteria were as follows: (A) secondary hypertension; (B) patients who had suffered acute infection, blood transfusion, trauma, or surgery within one month before admission; (C) patients with hematological diseases, rheumatic autoimmune system diseases, malignant tumors, and serious cardiovascular diseases; and (D) recent use of iron, folic acid, and other drugs affecting blood cell count. Therefore, 429 children with essential hypertension were enrolled in this study.

This study conformed to the principles of the Declaration of Helsinki. Each participant, as well as their parents and legal guardians, provided informed consent, and the study was approved by the ethical committee of the Capital Institute of Pediatrics, Beijing, China (No: SHERLL2019003).

### General clinical information and biochemical measurements

General clinical information included age, sex, height, weight, family history of hypertension, stage of hypertension, and body mass index (BMI) was calculated. Data were collected by experienced pediatricians. All hospitalized children fasted overnight for 8 h. In the morning, 2 ml of fasting venous blood was collected. The enzymatic method was used to measure blood glucose, the endpoint method was used to measure blood lipids, including triglyceride (TG), total cholesterol (TC), high-density lipoprotein cholesterol (HDL-C), and low-density lipoprotein cholesterol (LDL-C), and a colorimetric method was used to establish renal function, including creatinine, urea, and uric acid (UA). The above biochemical indicators were analyzed by an automatic biochemical analyzer (AU640, Olympius, Shizuoka, Japan). The plasma homocysteine (Hcy) level was measured using the Hcy test kit (Siemens Medical Diagnosis Co., Ltd., United States). C-reactive protein (CRP) was analyzed by automatic chemiluminescence immunoassay analyzer (I2000, Abbott Laboratories, United States).

### Complete blood count measurements

In the morning, 2 ml of fasting venous blood was drawn using a vacuum tube with ethylenediamine tetra-acetic acid. Blood specimens were stored at 4 °C and analyzed within 1 h of collection. To perform a complete blood count, an automated blood count instrument (XN-3000, Sysmex, Kobe, Hyogo, Japan) was used, and the RDW in this system typically ranged from 11.0% to 16.0%.

### Ambulatory blood pressure measurement

All children received 24 h ABPM (DMS-ABP, DM Software Inc., Beijing, China) (26). The blood pressure recording was set to measure and record blood pressure every 30 min, and the children were told to remain quiet and inactive while the device was inflated. To determine the overnight hours, sleep and wake times for each child were recorded and adjusted. We calculated the 24 h SBP and 24 h DBP based on these results. The measurement result was considered reliable when the measurement time exceeded 23 h and the measurement result exceeded 70% (27).

### Left ventricular hypertrophy and classification

We evaluated LVH by echocardiography. A Philips iE33 ultrasound system (Philips Healthcare, Bothell, WA, Unite States) was used to measure the LVIDd, IVST, LVPWT, LVEF, FS. The left ventricular mass (LVM) was determined using the Devereux formula (28) as follows:

$$\mathrm{LVM}=1.04\times 0.8\times \left[{\left(\mathrm{LVIDd}+\mathrm{IVST}+\mathrm{LVPWT}\right)}^{3}-\mathrm{LVIDd}{}^{3}\right]+0.6$$

The left ventricular mass index (LVMI) was calculated as follows:

$$\mathrm{LVMI}=\frac{\mathrm{LVM}}{\mathrm{heigh}t^{2.16}}$$

The relative wall thickness (RWT) was calculated as follows:

$$\mathrm{RWT}=\frac{\mathrm{LVST}+\mathrm{LVPWT}}{\mathrm{LVIDd}}$$

LVMI ≥ 45 g/m^2.16^ or RWT > 0.41 was diagnosed as LVH (29, 30). There were two groups of children, 114 with LVH (LVH group) and 315 without (non-LVH group).

### Statistical analysis

Shapiro–Wilk test was used to test the normality of continuous variables. Normally distributed variables were presented as the mean ± standard deviation (SD), and differences were compared with the independent *t*-test, non-normally distributed variables were expressed as the median (P_25_, P_75_), and the Mann–Whitney *U* test was used for comparisons. The chi-square test was used to compare categorical variables, which were represented by absolute number accompanied by a representative percentage. The non-parametric Spearman correlation was used to verify the association between RDW and LVMI, RWT, CRP, and RBC parameters. Multivariate logistic regression models were applied to the risk factor analysis for LVH in pediatric essential hypertension. The receiver operating characteristic (ROC) curve was used to evaluate the predictive value of RDW on LVH.

The statistical analysis was completed with SPSS statistical software version 26.0 (IBM Corp, Armonk, NY, United States). All tests were two sided, and *P* values < 0.05 were considered statistically significant.

## Results

### Clinical characteristics and biochemical parameters in LVH and non-LVH group

510 hypertensive children were admitted to the hospital for the first time throughout the research period but did not receive treatment. We excluded 81 cases, including 6 cases of secondary hypertension, 51 cases of acute infection, 12 cases of missing data, 11 cases of blood system diseases (10 cases of anemia, 1 case of thrombocytopenia), and 1 case who was < 6 years. Therefore, 429 children with essential hypertension (114 cases in the LVH group and 315 cases in the non-LVH group) were enrolled in our study.

The clinical characteristics and biochemical parameters of the subjects are presented in Table 1. This study included 429 patients (male, 78.6%) with a median age of 13.0 (12.0, 14.0) years. In the LVH group, the male ratio, age, BMI, hypertension stage 2 ratio, creatinine, UA, Hcy, and CRP were all considerably greater than those in the non-LVH group, although the HDL-C was significantly lower.

**Table 1:** Clinical characteristics and biochemical parameters in LVH and non-LVH group.

	non-LVH (*n* = 315)	LVH (*n* = 114)	*P*
**Clinical characteristics**			
Sex (male [%])	238 (75.6%)	99 (86.8%)	0.012
Age (years)	13.0 (11.0, 14.0)	13.0 (12.0, 14.0)	0.007
Family history of hypertension (*n* [%])	272 (86.3%)	103 (90.4%)	0.270
BMI (kg/m^2^)	26.14 (23.14, 29.41)	29.76 (27.14, 33.16)	<0.001
hypertension stage 2 (*n* [%])	149 (47.3%)	80 (70.2%)	<0.001
24 h SBP (mmHg), mean ± SD	127.5 ± 11.2	132.1 ± 11.3	<0.001
24 h DBP (mmHg), mean ± SD	72.2 ± 7.5	72.2 ± 7.7	0.990
**Biochemical parameters**			
TG (mmol/L)	1.14 (0.82, 1.59)	1.31 (0.90, 1.66)	0.198
TC (mmol/L), mean ± SD	4.00 ± 0.73	4.04 ± 0.72	0.623
HDL-C (mmol/L)	1.09 (0.94, 1.27)	1.01 (0.90, 1.17)	0.010
LDL-C (mmol/L), mean ± SD	2.61 ± 0.75	2.74 ± 0.71	0.106
Blood glucose (mmol/L)	4.48 (4.18, 4.77)	4.45 (4.11, 4.74)	0.424
Creatinine (μmol/L), mean ± SD	51.79 ± 11.86	55.29 ± 12.30	0.008
Urea (mmol/L)	4.38 (3.70, 5.12)	4.50 (3.71, 5.12)	0.514
UA (μmol/L)	424.0 (359.0, 487.0)	466.0 (415.0, 525.8)	<0.001
Hcy (μmol/L)	10.38 (8.17, 13.92)	11.44 (9.23, 14.55)	0.010
CRP (mg/L)	0.54 (0.48, 1.70)	0.81 (0.55, 2.13)	<0.001

non-LVH, none of left ventricular hypertrophy; LVH, left ventricular hypertrophy; BMI, body mass index; SBP, systolic blood pressure; DBP, diastolic blood pressure; TG, triglyceride; TC, total cholesterol; HDL-C, high-density lipoprotein cholesterol; LDL-C, low-density lipoprotein cholesterol; UA, uric acid; Hcy, homocysteine; CRP, C-reactive protein. Data are presented as median(P_25_, P_75_), except where otherwise indicated.

The results of 24 h ABPM revealed that the 24 h SBP of the LVH group was greater than that of the non-LVH group, while there was no significant difference in the 24 h DBP between the LVH group and non-LVH group.

### Echocardiography parameters in LVH and non-LVH group

The results of echocardiography showed that the mean LVMI and RWT of the 429 patients was 38.45 (33.74, 44.74) g/m^2.16^, and was 0.33 (0.30, 0.35). 114 children with hypertension (26.6%) had LVH; among them, 96 cases had elevated LVMI and normal RWT, demonstrating eccentric remodeling, 9 cases had normal LVMI and elevated RWT, demonstrating concentric remodeling, and 9 cases had elevated LVMI and RWT, demonstrating centripetal hypertrophy. Significantly greater than those in the non-LVH group were the LVM, LVMI, RWT, LVIDd, IVST, and LVPWT in the LVH group. Between the two groups, there was no discernible difference in left ventricular systolic function (LVEF and FS) (Table 2).

**Table 2:** Echocardiography parameters in LVH and non-LVH group.

	non-LVH (*n*** **=** **315)	LVH (*n*** **=** **114)	*P*
LVM (g)	111.00 (92.60, 128.00)	166.50 (146.75, 186.00)	< 0.001
LVMI (g/m^2.16^)	36.55 (32.30, 39.96)	49.27 (46.76, 53.46)	< 0.001
RWT	0.32 (0.29, 0.34)	0.34 (0.32, 0.38)	< 0.001
LVIDd (cm)	4.68 (4.39, 4.90)	5.19 (4.91, 5.39)	< 0.001
IVST (cm)	0.74 (0.66, 0.79)	0.86 (0.82, 0.93)	< 0.001
LVPWT (cm)	0.76 (0.69, 0.82)	0.89 (0.86, 0.97)	< 0.001
LVEF (%)	70.00 (66.30, 73.10)	69.45 (65.80, 72.70)	0.182
FS (%)	39.40 (36.60, 42.00)	39.30 (36.30, 42.03)	0.455

non-LVH, none of left ventricular hypertrophy; LVH, left ventricular hypertrophy; LVM, left ventricular mass; LVMI, left ventricular mass index; RWT, relative wall thickness; LVIDd, left ventricular internal dimension; IVST, interventricular septal thickness; LVPWT, left ventricular posterior wall thickness; LVEF, left ventricular ejection fraction; FS, fractional shortening. Data are presented as median (P_25_, P_75_).

### Complete blood count parameters in LVH and non-LVH group

The results of complete blood count showed that, compared with the non-LVH group, the RBC count and hematocrit level in the LVH group were significantly higher. Most importantly, the level of RDW in the LVH group was higher than that in the non-LVH group (13.0 [12.0, 13.0] vs. 12.4 [12.0, 13.0] %, *P* = 0.001). In other variables of complete blood count analysis, including white blood cells (WBCs), did not significantly differ between the LVH group and non-LVH group (Table 3).

**Table 3:** Complete blood count parameters in LVH and non-LVH group.

	non-LVH (*n*** **=** **315)	LVH (*n*** **=** **114)	*P*
RBC (10^12^/L), mean ± SD	5.02 ± 0.38	5.12 ± 0.36	0.017
HGB (g/L), mean ± SD	143 ± 12	145 ± 11	0.068
Hematocrit (%), mean ± SD	42.05 ± 3.19	42.90 ± 2.97	0.013
MCV (fl), mean ± SD	83.8 ± 3.8	83.8 ± 3.6	0.855
MCH (pg), mean ± SD	28.5 ± 1.6	28.4 ± 1.5	0.583
MCHC (g/L), mean ± SD	339.9 ± 9.7	338.5 ± 9.7	0.195
RDW (%)	12.4 (12.0, 13.0)	13.0 (12.0, 13.0)	0.001
Platelet (10^9^/L)	297 (264, 344)	295 (262, 333)	0.390
MPV (fl)	10.3 (9.9, 10.9)	10.4 (9.9, 11.0)	0.490
PDW (fl)	11.6 (10.9, 12.8)	11.9 (11.2, 13.1)	0.169
WBC (10^9^/L)	7.63 (6.46, 8.78)	7.38 (6.43, 8.62)	0.429
Lymphocyte (10^9^/L)	2.86 (2.46, 3.37)	2.84 (2.39, 3.47)	0.943
Neutrophils (10^9^/L)	3.99 (3.15, 4.99)	3.83 (3.27, 4.59)	0.511

non-LVH, none of left ventricular hypertrophy; LVH, left ventricular hypertrophy; RBC, red blood cell; HGB, hemoglobin; MCV, mean corpuscular volume; MCH, mean corpuscular hemoglobin; MCHC, mean corpuscular hemoglobin concentration; RDW, red blood cell distribution width; MPV, mean platelet volume; PDW, platelet distribution width; WBC, white blood cell. Data are presented as median(P_25_, P_75_), except where otherwise indicated.

### Correlation analysis of RDW

**Figure 1:**
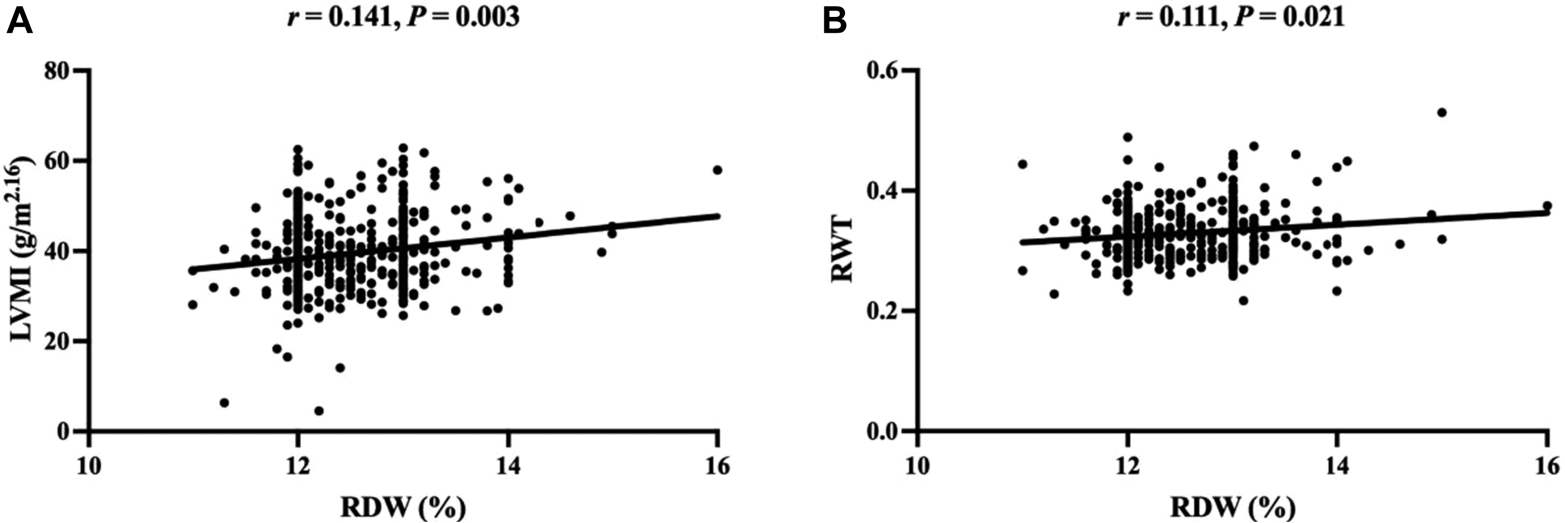
Scatter plot of correlation between RDW and LVMI **(A)**, RWT **(B)**. RDW, red blood cell distribution width; LVMI, left ventricular mass index; RWT, relative wall thickness.

As presented in Table 4, Spearman correlation analysis showed that RDW was positively correlated with CRP and RBC, but was negatively correlated with hemoglobin (HGB), mean corpuscular volume (MCV), mean corpuscular hemoglobin (MCH), and mean corpuscular hemoglobin concentration (MCHC). Most importantly, the RDW was positively correlated with LVMI and RWT (Figure 1).

**Table 4:** The correlations between RDW and clinical indices in pediatric essential hypertension.

Variable	r	*P*
CRP	0.186	< 0.001
LVMI	0.141	0.003
RWT	0.111	0.021
RBC	0.159	0.001
HGB	−0.198	< 0.001
MCV	−0.353	< 0.001
MCH	−0.471	< 0.001
MCHC	−0.373	< 0.001

CRP, C-reactive protein; LVMI, left ventricular mass index; RWT, relative wall thickness; RBC, red blood cell; HGB, hemoglobin; MCV, mean corpuscular volume; MCH, mean corpuscular hemoglobin; MCHC, mean corpuscular hemoglobin concentration.

### Multivariate logistic regression analysis of LVH in pediatric essential hypertension

As seen from the multivariate logistic regression analysis (Table 5 unadjusted), the RDW levels in pediatric essential hypertension were independently associated with the occurrence of LVH. Moreover, after adjusting for different influencing factors, the level of RDW was still closely related to the occurrence of LVH (Table 5 adjusted). Male sex and BMI were also considered independent risk factors for LVH.

**Table 5:** Multivariate logistic regression analysis of LVH in pediatric essential hypertension.

Risk factors	Unadjusted				Adjusted			
	OR	95% CI		*P*	OR	95% CI		*P*
Male sex	2.135	1.171	3.894	0.013	2.158	1.021	4.564	0.044
BMI	1.183	1.125	1.244	<0.001	1.169	1.098	1.245	<0.001
RDW	1.866	1.349	2.580	<0.001	1.946	1.324	2.861	0.001

OR: odd ratio, CI: confidence interval; BMI, body mass index; RDW, red blood cell distribution width. Adjusted for age, stage of hypertension, 24 h SBP, HDL-C, Creatinine, UA, Hcy, CRP, RBC, Hematocrit.

### Predictive value of RDW for LVH

**Figure 2:**
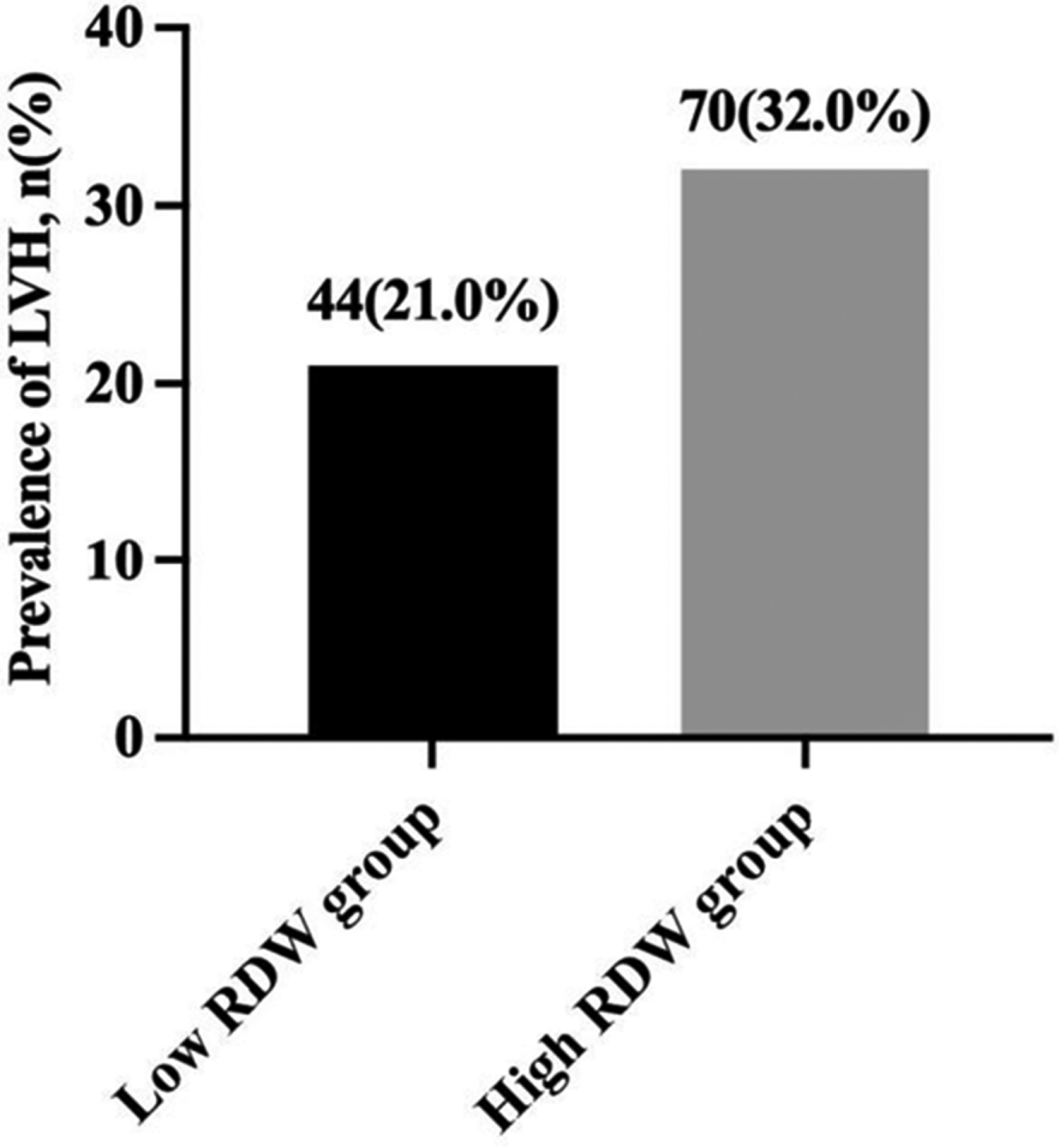
Prevalence of LVH in children with essential hypertension according to the dichotomies of RDW. LVH, left ventricular hypertrophy; RDW, red blood cell distribution width.

According to the dichotomies of RDW, all children were split into two groups: the low RDW group (RDW ≤ 12.40%, *n* = 210) and the high RDW group (RDW > 12.40%, *n* = 219). The prevalence of LVH in children with essential hypertension was 21.0% in low RDW group and 32.0% in high RDW group (*χ*^2^ = 6.661, *P* < 0.05) (Figure 2).

A ROC curve was drawn according to the level of RDW and LVH in pediatric essential hypertension. The area under the curve (AUC) was 0.608 (95% CI: 0.546–0.670, *P* = 0.001), and the cut-off value was 12.75%. The sensitivity and specificity of RDW in predicting LVH status in children with hypertension were 55.3% and 65.1%, respectively. The AUC predicted by RDW level for centripetal hypertrophy was 0.700 (95% CI: 0.541–0.859, *P* < 0.05), the critical value was 12.65%, the sensitivity was 77.8%, and the specificity was 56.9%. The RDW level had a low predictive value for eccentric remodeling and concentric remodeling (Supplementary Figure S1).

## Discussion

This study provides the first data on the association between RDW levels and LVH in untreated pediatric essential hypertension. The data showed that RDW levels were significantly increased in children with hypertension in the LVH group compared to those in the non-LVH group. Moreover, the RDW levels were significantly positively correlated with LVMI and RWT levels in children with hypertension. Additionally, RDW was found to be an independent influencing factor of LVH in children with hypertension, and may be a predictor of LVH in untreated essential children with hypertension.

The rising prevalence of hypertension in children and adolescents has emerged as a major public health issue. Most children with essential hypertension have no obvious clinical symptoms, which makes the early diagnosis of hypertension difficult. Some children with hypertension show early changes in target organs at diagnosis (25). According to previous research, the prevalence of LVH in pediatric essential hypertension ranges from 19% to 41.6% (28, 31). In this study, 26.6% of children with hypertension were diagnosed with LVH during the first visit, indicating that subclinical cardiac damage occurs before the first visit. The RDW is a simple hematological parameter that is commonly obtained in a standard complete blood count and is used to help differentiate between different types of anemia. Many adult cross-sectional studies have confirmed that RDW is closely related to target organ damage, such as to the heart and kidney, in patients with hypertension (23, 24, 32). Recently, several large prospective studies have focused on the value of RDW in hypertension incidence and cardiovascular events. In an observational study of 240,477 healthy UK volunteers over 9 years, RDW was predictive of hypertensive events after adjustment for multiple factors (12). Similarly, Seo et al. studied 124,261 Korean participants without hypertension, and showed that the incidence of hypertension was significantly higher in participants with higher levels of RDW than in those with lower levels of RDW after 11 years of follow-up, which was significantly and independently related with the occurrence of hypertension (13). Moreover, researchers from Turkey followed 1,202 adults with newly diagnosed hypertension for up to 7 years. During the entire follow-up period, the incidence of cardiovascular death was significantly higher in patients with high RDW levels compared with those with low levels of RDW. Therefore, the RDW was an independent predictor of long-term cardiovascular events in patients with hypertension (33). However, research on the relationship between RDW and hypertension in children is limited. Hu et al. studied 80 children with orthostatic hypertension and 51 healthy children with age and sex matching in China, and discovered that the RDW levels in children with orthostatic hypertension were considerably greater than those in the healthy children. They also found that the increase in RDW was a risk factor for orthostatic hypertension in children after adjusting for other factors (34). In this study, the RDW levels in the LVH group were significantly higher than those in the non-LVH group. The RDW levels were significantly correlated with LVH in children with hypertension, which was consistent with studies in adults (23). ROC curve analysis revealed that RDW had better predictive value for centripetal hypertrophy in the classification of LVH, indicating that RDW had better predictive value for LVH children with both LVMI and RWT increased.

Essential hypertension is a complex multifactorial disease. The exact mechanisms of RDW, essential hypertension and LVH remain unclear, previous research has indicated that the mechanisms may be closely related to the RAAS activation and inflammatory reaction. The RAAS exists in human blood circulation and local tissues and plays a significant role in regulating vascular tension, water electrolyte balance, and heart and vascular remodeling through angiotensin II. The activation of the RAAS can lead to vasoconstriction, stimulate the growth of vascular smooth muscle cells, and cause myocardial hypertrophy, ventricular remodeling, myocardial ischemia, coronary blood flow reduction, water and sodium retention, and a significant increase in blood pressure (35). Previous studies by our team have found that angiotensin II is not only a trigger for LVH in children with hypertension, but is also involved in the disease progression of LVH (36). Erythropoietin (EPO) is a key regulator of erythropoiesis. Studies have confirmed that the increase in angiotensin II can directly promote the secretion of EPO (16), which subsequently promotes the premature release of immature erythrocytes from the bone marrow into the blood. This promotes abnormal erythropoiesis and increases the production of incomplete mature erythrocytes, leading to elevated RDW levels (17). In this study, Spearman correlation analysis showed that the RDW level was positively correlated with RBC, and negatively correlated with HGB and RBC-volume parameters. This suggests that the increase in RBCs with a high RDW may be dominated by the increase in the number of small-volume RBCs. Increasing evidence shows that inflammation is directly involved in the increase in blood pressure and the occurrence of target organ damage in children (37–39). The maturation of erythrocytes may be impacted by the effects of inflammatory cytokines on the pace of bone marrow hematopoiesis, erythropoietin production, and iron metabolism processes (40, 41). Moreover, the accumulation of immature erythrocytes changes the volume and size of erythrocytes, increasing the RDW (42, 43). In this study, the CRP level in the LVH group was significantly higher than that in the non-LVH group. Furthermore, the RDW was positively correlated with CRP in children with hypertension, suggesting that inflammation plays a role in the pathogenesis of LVH and RDW elevation in children with hypertension.

There are some limitations to this study. First, the causal relationship between RDW and LVH could not be determined due to the cross-sectional design. Second, while we found a link between elevated RDW and LVH, we were unable to pinpoint the exact mechanism by which this link was formed. Finally, the sample size for this study, which is based on data from Chinese children with hypertension in a single center, is quite tiny.

## Conclusion

In conclusion, our results showed that RDW levels were higher in children with hypertension in the LVH group than in the non-LVH group, and increased RDW levels were independently related with LVH in children with hypertension. The RDW is easy to obtain and operable in clinical diagnosis and treatment activities, and may serve as a predictor of LVH in untreated pediatric essential hypertension.

## Data Availability

The original contributions presented in the study are included in the article/Supplementary material, further inquiries can be directed to the corresponding author/s.
