# Supplementary material for: Association between red blood cell distribution width and left ventricular hypertrophy in pediatric essential hypertension
Source: Front Pediatr. 2023 Feb 2;11:1088535. doi: 10.3389/fped.2023.1088535 (PMC9932496; doi:10.3389/fped.2023.1088535)
Supplement: Supplementary file 1 [file Table2.docx]

Supplementary Material

# Supplementary Figures

## Supplementary Figures

| 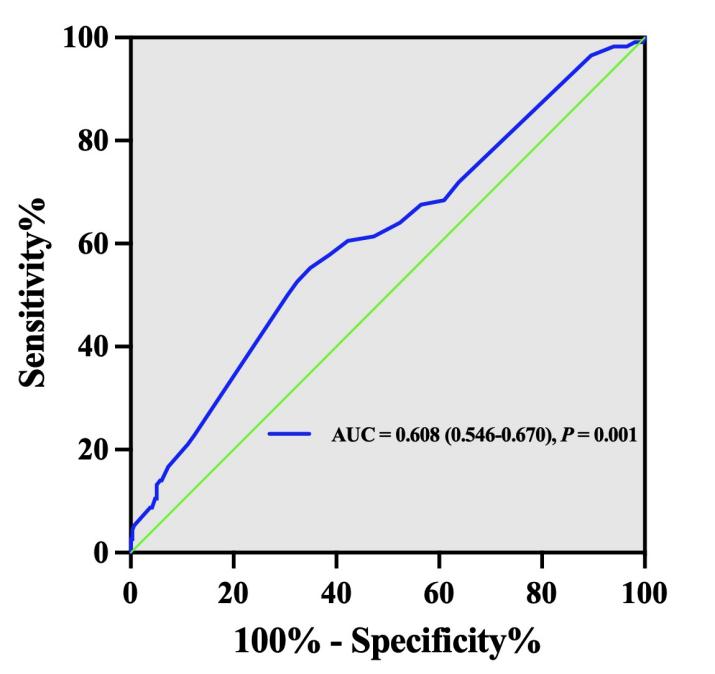  **A** | 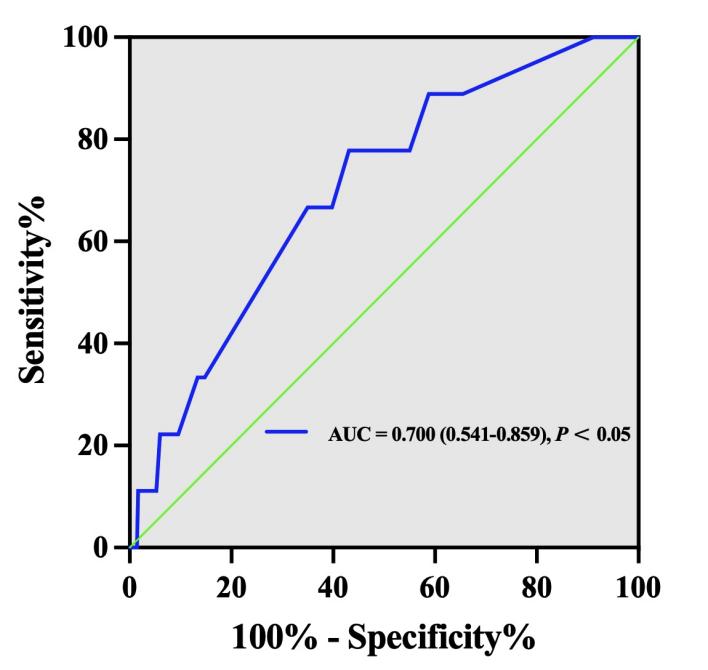  **B** |
| --- | --- |

**Supplementary Figure 1.** ROC curve analysis using RDW level for prediction of LVH **(A)** and centripetal hypertrophy **(B)** in pediatric essential hypertension.

(AUC, area under curve; ROC, receiver operating characteristic; RDW, red blood cell distribution width; LVH, left ventricular hypertrophy)
